# Audio-Biofeedback training for posture and balance in Patients with Parkinson's disease

**DOI:** 10.1186/1743-0003-8-35

**Published:** 2011-06-21

**Authors:** Anat Mirelman, Talia Herman, Simone Nicolai, Agnes Zijlstra, Wiebren Zijlstra, Clemens Becker, Lorenzo Chiari, Jeffrey M Hausdorff

**Affiliations:** 1 Laboratory for Gait and Neurodynamics, Tel Aviv Sourasky Medical Center, Tel Aviv, Israel; 2 Robert-Bosch-Hospital, Department of Clinical Gerontology, Stuttgart, Germany; 3 Center for Human Movement Sciences, University Medical Center Groningen, University of Groningen, Groningen, The Netherlands; 4 Department of Electronics, Computer Science & Systems, Università di Bologna, Bologna, Italy; 5 Department of Physical Therapy, Ben Gurion University, Beer Sheba, Israel; 6 Department of Physical Therapy, Sackler Faculty of Medicine, Tel Aviv University, Tel Aviv, Israel

**Keywords:** Intervention, mobility, neurodegenerative disease, postural control, posture, Parkinson's disease

## Abstract

**Background:**

Patients with Parkinson's disease (PD) suffer from dysrhythmic and disturbed gait, impaired balance, and decreased postural responses. These alterations lead to falls, especially as the disease progresses. Based on the observation that postural control improved in patients with vestibular dysfunction after audio-biofeedback training, we tested the feasibility and effects of this training modality in patients with PD.

**Methods:**

Seven patients with PD were included in a pilot study comprised of a six weeks intervention program. The training was individualized to each patient's needs and was delivered using an audio-biofeedback (ABF) system with headphones. The training was focused on improving posture, sit-to-stand abilities, and dynamic balance in various positions. Non-parametric statistics were used to evaluate training effects.

**Results:**

The ABF system was well accepted by all participants with no adverse events reported. Patients declared high satisfaction with the training. A significant improvement of balance, as assessed by the Berg Balance Scale, was observed (improvement of 3% p = 0.032), and a trend in the Timed up and go test (improvement of 11%; p = 0.07) was also seen. In addition, the training appeared to have a positive influence on psychosocial aspects of the disease as assessed by the Parkinson's disease quality of life questionnaire (PDQ-39) and the level of depression as assessed by the Geriatric Depression Scale.

**Conclusions:**

This is, to our knowledge, the first report demonstrating that audio-biofeedback training for patients with PD is feasible and is associated with improvements of balance and several psychosocial aspects.

## Introduction

Postural instability, gait disturbances and falls are a leading cause of morbidity and mortality among older adults [1-6], especially among patients suffering from a neurodegenerative disease like Parkinson's disease (PD). Because of the tremendous impact of falls on functional independence, health care economics, social function and health-related quality of life, much effort has been dedicated to identify the physiologic factors that contribute to fall risk. This includes prospectively monitoring those individuals with an increased fall risk and developing interventions for improving balance control and reducing falls [1-6].

In PD, postural instability and falls usually occur during the more advanced stages of the disease and are among the most disabling motor symptoms [7]. These deficits are most probably due to an accumulation of factors such as stooped posture and decreased postural reflexes, hypokinesia, diminished and fragmented postural responses, and impaired cognitive ability [8-11]. While much is known at the present about the multi-factorial nature of gait disturbances and falls in PD, there are still many questions regarding the best therapeutic means of improving these impairments and thus reducing fall risk. Specific forms of exercise have been recommended as elements of fall-prevention programs for older adults, for example, aerobic-type exercises and exercises that target balance, strength and gait are common elements of multi-factorial fall prevention interventions [12-14]. However, typically, these interventions report a reduction in fall risk by only 10% to 20% [15,16] and are not yet optimal. Moreover, these programs do not always address the specific needs for parkinsonian symptoms that give rise to poor balance and gait.

The use of biofeedback has been offered in the past as an instrument for training that enables an individual to learn how to change physiological activity or behavior for the purposes of improving performance. Biofeedback training of balance and posture has shown to be effective for posture control in adolescents with scoliosis [17] and has decreased fall rate in elderly patients with peripheral neuropathy [18]. In patients with bilateral vestibular loss [19], biofeedback training was also found useful in enhancing postural stability even under challenging standing conditions (e.g., tandem walking), beyond the effect of practice alone [19-21]. Based on these previous studies, we hypothesized that deficits in postural control in patients with PD can be positively influenced by Audio Bio-Feedback (ABF) -based dynamic balance training. The aims of this study were to investigate the manner and tasks in which the ABF system can be used to enhance postural control in PD, to explore the feasibility of using an ABF system for training stability of those patients, and to preliminary assess the usability and efficacy of a new ABF-based paradigm on a small group of patients with PD.

## Methods

### Participants and Design

**Figure 1:**
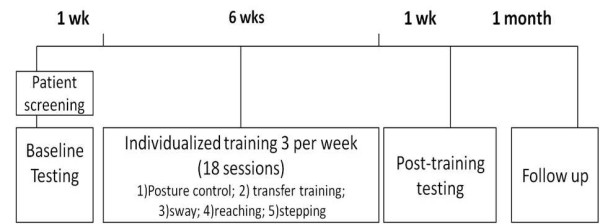
**A schema of the study procedure**.

In this pilot intervention study, a repeated measures design with a six week intervention program was used. We aimed to improve posture, static and dynamic balance and activities of daily living (ADLs) such as rising from sit to stand and reaching. Seven patients with PD (mean age 71.4 years, range 59-85 years; 1 female, 6 males) were recruited from the Movement Disorders Unit at Tel Aviv Sourasky Medical Center (TASMC) and enrolled in this intervention study. Inclusion criteria included a diagnosis of idiopathic PD (at least 2 years), the ability to walk independently without a walking aid, and the absence of serious co-morbidities that could impact gait or balance. Patients were excluded if they suffered from major depression, Mini Mental Status Examination [22] score <24, had clinically significant hearing problems which may hinder their ability to hear the feedback sound provided, or were medically unstable. The assessments were performed at baseline (within one week before the beginning of the intervention), immediately post training (within one week after the last training session) and four weeks after the completion of the training (follow-up assessment). Each training session lasted approximately 45 minutes (see Figure 1) and was provided by a physical therapist three times a week at the Laboratory for Gait and Neurodynamics at TASMC. Five patients also received several training sessions (up to 3 training sessions) in their home to explore the possibility for future independent home training with the ABF system. The home sessions were performed in the last 2 weeks of the training, when patients were already familiar with the system and could attempt to use it independently with only the supervision of the therapist. The study was approved by the ethical committee of the local medical center. Written consent form was provided by all participants.

### Audio Bio-Feedback (ABF) system

**Figure 2:**
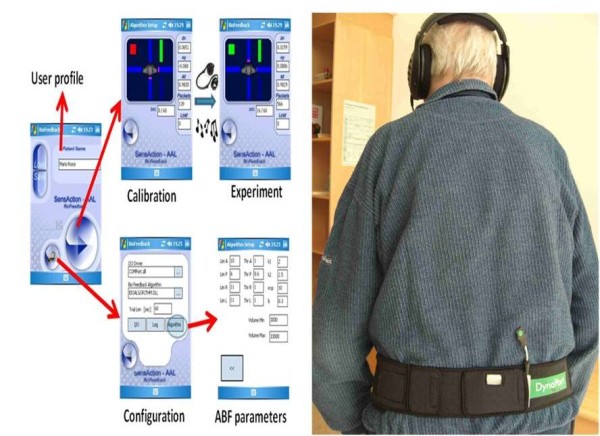
**The ABF device used in this study**. The device is worn on the patient's lower back and is attached to headphones by which he hears the auditory feedback. On the right is an example of the training configuration as presented on the PDA.

The ABF system that was used in this study was developed as a prototype that emanated from the SensAction-AAL project [23]. The goal of the Sensaction-AAL project was to develop a home-based monitoring and intervention system that would provide both audio biofeedback for training but will also be able to monitor activities and detect falls in the elderly. The small-sized and light-weighted device contains tri-axial accelerometers and gyroscopes and was attached to the lower back using a velcro belt between the levels of L2-L5 vertebras, without hindering the subject during exercise. The ABF system was connected to a personal digital assistant (PDA) via Bluetooth (see Figure 2). Headphones were attached to the PDA through which the patient was able to hear the provided feedback. The patient received an auditory feedback which was modulated in frequency and amplitude by the participants movement and change of body orientation (trunk accelerations) in both the medio-lateral (ML) and anterior-posterior (AP) directions (2-D). The modulation of the sound was tied to one or more target zones (defined by a pattern of trunk inclination and local accelerations) which were adaptively estimated during a short initial calibration phase in the beginning of each training session [19,24]. Two different types of feedback were used: (a) negative feedback, a sound outside of the target zone, for example, posture correction during standing; in the form of a higher pitch sound was provided if the subject returned to a mal aligned posture from the desired erect position), (b) positive feedback, a sound inside the target zone, in which the device was silent when the movement was correct, for example when the subject was able to maintain a challenging position, such as standing with one leg on a stool, without losing balance. The target region was calibrated individually prior to each exercise to predefine the desired range of motion.

### Training Protocol

The training program followed three major objectives: (1) to improve body posture and static balance (2) to improve dynamic balance, and (3) to improve activities of daily living (ADLs), i.e., sit to stand abilities and reaching. The intervention included a variety of exercises from six categories of posture and balance with increasing difficulty and complexity. These included: (1) static posture control-achieving better upright position while sitting and in standing (improving upper limb and shoulder girdle range of motion and endurance while maintaining the predefined positions), (2) transfers (improving sit-to-stand and stand-to-sit activities), (3) sway (quiet standing, weight shifting to all directions, loading/unloading, additional upper body movements, differences in the base of support; e.g., foot position, foam), (4) reaching in different directions with movement of the trunk, (5) stepping in different directions and onto steps in different heights. Both reaching and stepping exercises were sometimes performed with additional upper body movements, and 6) obstacle clearance.

Every training session included different exercises from each category. Sessions were individualized to fit each patient's specific needs and were based on performance in the previous session, gradually progressing with intensity and complexity. For example, a session could begin with a posture task in standing with the patient trying to maintain an erect upright posture; this would then progress to a reaching exercise in different directions while the patient would still be required to maintain the upright posture when returning to the standing position after reaching his target. A possible progression could then include a stepping exercise over obstacles of different heights while maintaining minimal sway after the obstacle was negotiated. The system provided feedback during the exercises. The order of the exercises within the training sessions was pre-defined for all participants, but the progression within the categories was determined individually based on the patient's ability and needs, continuously adjusting and challenging the patient. The rational for this training program was based on motor learning paradigms aimed at providing demanding tasks for the patient and allowing knowledge of performance and results to enhance practice and learning [25]. Mean exercise duration was between 2 and 3 minutes depending on the patient's ability, tolerance and endurance, with total net training time of 30-45 minutes in each session.

### Assessments

Assessments included standardized tests of balance and, postural control as well as ADL's to evaluate the effects of training. Balance tests that were used included: 1) The Berg-Balance Scale (BBS) which consists of 14 different balance tasks such as standing, reaching, bending, and transferring abilities, and has an overall score range from 0 (severely impaired) to 56 points (excellent) [26]; 2) The Timed Up-and-Go (TUG) test was used to assess the ability to perform sequence movements of functional mobility. Patients were instructed to stand up from a chair, walk for a distance of 3 meters at comfortable speed, turn, walk back, and sit down on the chair [27]. Time was measured with a stopwatch and the average of two trials was taken; 3) the 5 chair rise (5CR) test was used to assess the ability to perform sit-to-stand and stand-to-sit transfers. Patients were instructed to stand up and sit down five times as fast as possible starting in the sitting position and stopping after sitting down the fifth time [28]. Here too, the average duration of two trials was taken. The scores of the sub items and the total score of the Parkinson's disease questionnaire (PDQ-39) were used to determine health-related quality of life. The eight sub items of this questionnaire cover mobility, activity of daily living, emotional well-being, stigma, social support, cognitive impairment, communication, and bodily discomfort [29].

To quantify extra-pyramidal signs and disease severity, the Unified Parkinson's Disease Rating Scale (UPDRS) was used [7] and to assess the confidence in daily activities and the level of fear of falling, we used the Activities-specific Balance Confidence (ABC) scale [30]. Finally, The Geriatric Depression Scale short form (GDS-15) was used for the assessment of emotional wellbeing and depressive mood [31].

### Data analysis

Descriptive statistics were used to evaluate the effects of training on balance and postural control. Average, standard deviations and ranges were extracted as well as the percent change after training and at follow up from the initial baseline evaluation. Training effects (pre vs. post and pre vs. follow-up) were evaluated using the Wilcoxon signed rank test and were assumed to be significant at p < 0.05 (two-sided). All analyses were conducted with SPSS version 16 software (SPSS Inc., Chicago, IL, USA).

## Results

All participants completed the 18 training sessions and all evaluations and reported generally high satisfaction from the program. Demographic and clinical details of the participants are summarized in Table 1. No adverse events were reported either during training in the gait laboratory or in the participants home's. All patients subjectively reported that both sound and exercises using the ABF device were easy to understand and were agreeable, the device was light weight, and was not cumbersome. Participants reported that the training was generally interesting and challenging in regards to the motor and balance demands. Three patients also mentioned that the training required concentration and attention abilities in order to perform the task presented successfully.

**Table 1:** Patients characteristics

N = 7	Mean	SD	Range
Age [yrs]	71.3	8.3	59-85
Height [cm]	171	5.6	163.0-177.0
Weight [kg]	70.85	10.1	58.0-90.0
BMI [kg/m^2^]	25.1	4.5	21.7-33.9
MOCA [0-30]	21.4	1.4	20-24
Age of disease onset [yrs]	61.0	2.6	47-70
Duration of disease [yrs]	10.3	5.7	4-19
Hoehn and Yahr	2.5	0.5	2-3

BMI - Body Mass Index; M0CA - Montreal Cognitive Assessment, 30 = best value

Positive trends were observed in all measures of balance control in response to the training when subjects were assessed after the conclusion of the 6 weeks program. The TUG scores improved by 11%; (p = 0.07), time to perform 5 sit-to-stand improved by 7.3% (p = 0.09) and the BBS significantly improved by 3% (p = 0.032) (Table 2). Improvements in the BBS were mainly observed in items 12 and 13 (stepping onto a step and standing in tandem). Trends for improvements were also observed in the UPDRS rating scale (3.3%) with specific changes observed in the pull test (item # 29) in 5 out of the 7 patients at post training; this task was trained during the sessions and reflects a training specific change. Patients scored less (better) on the GDS (p = 0.05) and PDQ-39 scales, which suggests less depressive symptoms and higher quality of life (Table 2), however, there was no change in the perception of fear of falling (as measured by the ABC) as a result of the training.

**Table 2:** Immediate and long term training effects

Measures	Pre training	Post training	Follow up
Berg Balance test	49.0 ± 7.2 (35-55)	50.4 ± 6.7 (37-55)*	49.6 ± 9.2 (30-55)
Timed Up & Go (sec)	13.2 ± 4.1 (9.4-20.0)	11.7 ± 2.9 (9.2-17.1)	10.8 ± 2.4 (9.0-16.1)*
5 Chair Rise Test (sec)	16.6 ± 3.4 (14.3-21.4)	15.3 ± 1.0 (12.2-16.8)	N/A
UPDRS (part III)	25.3 ± 11.7 (12-48)	24.4 ± 10.6 (12-45)	23.4 ± 10.4 (12-44)
Posture (UPDRS item 28)	2.3 ± 0.6 (1-3)	2.2 ± 0.7 (1-3)	2.2 ± 0.7 (1-3)
Activities-specific Balance Confidence Scale (%)	73.2 ± 15.4 (49.8-97.5)	73.3 ± 15.9 (49.4-100)	73.7 ± 18.9 (40.9-100)
Geriatric Depression Scale	5.8 ± 5.0 (1-13)	3.8 ± 3.5 (0-10)	6.1 ± 5.3 (0-14)
PDQ-39			
Total score	33.4 ± 18.7 (15.1-62.5)	31.7 ± 18.5(12.3-58)	36.8 ± 17.5(16.1-51.6)
*Mobility index*	41.8 ± 19.9 (12.5-67.5)	40 ± 17.3 (12.5-70)	37.5 ± 14.9 (12.5-50)*
*ADL index*	48.2 ± 20.4 (20.8-70.8)	46.4 ± 17.6 (20.8-75)	46.6 ± 22.5 (20.8-75)
*Cognitive index*	39.5 ± 27.6 (6.2-75)	26.8 ± 15.6 (6.2-50)*	33.7 ± 20.5 (6.2-62.5)

Values are average ± SD (range); ABC - Activities-specific Balance Confidence, 0-100%, 100% = best; ADL, Activities of daily living, 0-100 points, 0 = best; BBS, Berg Balance Scale, 0-56 points, 56 = best; 5CR, five chair rise test; GDS, Geriatric Depression Scale, 0-15, higher = worst; TUG, Timed up-and-go test; UPDRS, Unified Parkinson's Disease Rating Scale, higher = worse; Total of the PDQ-39, higher = worst; Domains relevant to the training were also investigated separately. * p < 0.05 (at pre vs. post; at follow up vs. pre).

Changes in the TUG, BBS and UPDRS scores were maintained at follow-up and some measures even continued to improve compared to baseline (recall Table 2). Interestingly, there was deterioration in the PDQ-39 and GDS scores at follow-up from those measured immediately post training, however scores on the PDQ-39 were still better than at pre-training values.

## Discussion

To our knowledge, this is the first intervention trial using an ABF system for training posture and balance in patients with PD. In this pilot study, we demonstrated that ABF training in patients with PD is feasible and that it appears to be well accepted. Adherence to the training protocol was high with no attrition. All patients also reported satisfaction and enjoyment during the training program while the therapist commented on the ease of use of the device. Some of the training sessions were conducted in the patients' home-environment with the rationale that behavior and performance may be altered in a clinical setting with unfamiliar surroundings and that training in the home could address the particular needs of each patient. The sessions at home were similar to the lab sessions in the provided exercise program and tasks performed. Patients commented that they felt comfortable during the home sessions and that they could foresee a need for such training in the future.

This training program demonstrated some potential therapeutic effects on postural control and psychosocial aspects of the disease. Small, but positive changes were observed in the BBS, 5 chair rise test, TUG and the pull test of the UPDRS rating scale. Components of these tasks were trained during the intervention and therefore, these effects could be considered a result of task specific training. Although statistically significant, the improvements on the BBS revealed only a mild change in actual function. This may be due to the fact that the patients had relatively high scores at baseline suggesting that the measure may not have been sensitive enough to detect minor changes in balance tasks. Some of these improvements were also observed at follow-up demonstrating initial support for retention of the effects of ABF training even in the presence of neurodegeneration.

Patients also reported improved mood after training however, without a control group, it is difficult to know if the improvement should be attributed to the participation in this research study and its weekly routine, or if this was a beneficial by-product of the ABF training. Interestingly, the sub items that were affected by the training on the quality of life questionnaire (PDQ-39) were mobility, ADL and cognition, which are all consistent with the specific training goals and the particular training effects. Although scores on the Activities-specific Balance Confidence scale (ABC) did not change, anecdotally, patients described that they were able to move more freely, with less assistance and more confidence after the training. Once more, this finding could be attributed to the insufficient sensitivity of the ABC as the sections that were scored low initially on this scale were not addressed in this training protocol.

A key limitation of this study is the small sample size. The present study aimed to explore if this training method is feasible for patients with PD. As such, the findings are encouraging. Future studies should include a larger sample of patients and compare them to an active control group. Training with the ABF device teaches participants new strategies of movement that could be applied in real life situations. In this sense, the ABF may have an advantage over other technologies used in PD such as external cueing, by enhancing motor learning through feedback on knowledge of performance and knowledge of results. Although, there is evidence in the literature on the positive effects of cueing strategies on gait in PD [32-34], gait training with the ABF has yet to be examined. Further studies are needed to look at the possibility of using ABF for independent, home training, and specifically for the purpose of improving *gait *in PD. The findings of our study should also encourage therapists to perform ABF-based physical training in other age-associated disorders such as elderly with higher level gait disorders and older adults with high fall risk or with Mild Cognitive Impairment.

In conclusion, the results presented here demonstrate that ABF-based physical training for posture and balance in PD is feasible and associated with quantitative improvements. This may be viewed as a promising first step to implement home-based training strategies for patients with PD, a cohort which does not yet have sufficient therapeutic options for improving postural instability and alleviating gait disturbances.

## Competing interests

The authors declare that they have no competing interests.

## Authors' contributions

WZ, CB, LC and JMH participated in the conceptualization and development of the ABF device and contributed to data analysis. AM, TH, NS and AZ formulated the rehabilitation paradigm and training protocol. AM and TH were the main contributors in the acquisition of the data, analysis and interpretation of the clinical findings and manuscript preparation. All authors revised and approved the current version of the manuscript.
